# A Case of Personality and Behavioral Changes with Frontotemporal and Cerebellar Atrophy on MRI with Corresponding Hypometabolism on FDG-PET

**DOI:** 10.1155/2024/2863662

**Published:** 2024-08-19

**Authors:** Megan Selig, Gloria Lee, Brian Lebowitz, Dinko Franceschi, Nicole Absar

**Affiliations:** ^1^ Department of Neuropsychiatry Stony Brook University Renaissance School of Medicine, Stony Brook, NY, USA; ^2^ Neurocognitive Clinic Mass General Brigham—Martha's Vineyard Hospital, Martha's Vineyard, MA, USA

## Abstract

**Background:**

The differential diagnosis of a patient with cognitive, behavioral, and motor symptoms is broad. There is much overlap between neurocognitive disorders due to frontotemporal dementia and other subcortical dementia. A less known diagnosis, cerebellar cognitive affective syndrome (CCAS), should also be considered. *Case History*. A 29-year-old female presented with ataxia and left-sided weakness. CSF showed oligoclonal bands, and MRI showed multiple white matter lesions with some atrophy. She was diagnosed with multiple sclerosis (MS). At age 35, she developed frontal lobe symptoms and executive dysfunction; she was diagnosed with MS with bipolar disorder. Neuropsychological evaluation at that time showed significant deficits in multiple cognitive domains. Subsequent MRI showed progressive frontotemporal atrophy, and FDG-PET uncovered hypometabolism in the frontotemporal lobes and cerebellum. At age 38, her behavior worsened with aggression, and she was started on olanzapine. She responded well with decreased agitation and improved motivation and attention. Compared with previous scans, most recent MRI and FDG-PET showed interval increase in cerebellar atrophy with increase in hypometabolism in the cerebellum, respectively.

**Conclusion:**

Based on cerebellar, affective, and subcortical cognitive examination findings, our diagnosis is probable CCAS. The cerebellum should be considered as a possible etiology of frontal subcortical cognitive impairment.

## 1. Introduction

The differential diagnosis for a patient with cognitive, behavioral, and motor symptoms is broad. There is much overlap between neurocognitive disorders due to frontotemporal dementia (FTD) and other subcortical dementias. As an example, psychiatric misdiagnoses may occur in almost 50% of behavioral variant of FTD (bvFTD) patients, especially in those with certain clinical symptoms such as disinhibition, loss of empathy, or apathy [1]. Neurological and neuropsychiatric conditions often have varying presentations, making it difficult to come to a final diagnosis in patients with these symptoms. Here, we present the case of a 29-year-old female with atypical symptoms of cognitive impairment in addition to psychiatric symptoms. She had multiple diagnoses including multiple sclerosis (MS), bipolar disorder, and FTD, ultimately meeting the criteria for cerebellar cognitive affective syndrome (CCAS). This case illustrates the complexity of symptoms that cerebellar syndromes can present with and the importance of consideration of a broad differential in complex cases, including cerebellar syndromes.

## 2. Case History

Our patient is a 29-year-old female graduate of nursing school who presented with vertigo, ataxia, and left-sided weakness. Her CSF showed oligoclonal bands, and MRI showed multiple white matter lesions in the subcortical, periventricular, and deep white matter of both the cerebral hemispheres, the genu of corpus callosum, and the left middle cerebellar peduncle with some atrophy, leading to a diagnosis of MS. She was treated with a methylprednisolone infusion and received multiple treatments over the subsequent years for MS flares. Her MS was determined to be the relapsing–remitting type. At the age of 30, she had a positive JCV index of 0.89. Then, at age 32, she became pregnant and had a C-section prior to being started on rituximab. MRI at that age showed 32 new lesions with two new nonenhancing lesions in the right frontal centrum semiovale and a new lesion in the left cerebellar white matter. Later that year, she went to the psychiatric emergency room for depression with symptoms including anorexia, crying spells, and decreased energy, and she was started on sertraline before being switched to bupropion. Repeat MRIs continued to show white matter lesions in areas including the cerebellar peduncles. She continued to have MS symptoms, now including vertigo and weakness. In addition, she started to report emotional instability, for which she was placed on fluoxetine, and she took a leave of absence due to decline in cognition. Since age 33, the patient has not had any more new lesions on MRI FLAIR images.

At age 35, she developed frontal lobe symptoms and executive dysfunction and was admitted to the hospital for a manic episode, where she was diagnosed with MS with bipolar disorder. At that time, her symptoms included increased energy, emotional lability, grandiosity, impulsiveness, and reckless spending. Neuropsychological evaluation showed significant deficits in multiple cognitive domains, and subsequent MRI showed progressive frontotemporal atrophy, which was noted over the past 2 years when reviewed more closely. Following MRI, the patient had an FDG-PET, which uncovered hypometabolism in the frontotemporal lobes and cerebellum. CSF, EEG, and lab workup were consistent with possible autoimmune encephalitis, and intravenous immunoglobulin was administered with only partial and temporary improvement.

Over the following 2 years, the patient had repeat FDG-PET scans, which continued to show hypometabolism predominately in the frontotemporal lobes and were read as favoring frontotemporal dementia. At the age of 37, the patient was admitted to the hospital with catatonia, demonstrating echolalia and severe emotional distress, and neuropsychological evaluation was limited due to difficulty in participation from the patient. Monoclonal bands in lumbar puncture at this time disappeared, putting the history of MS into question. The patient was tried on memantine, ramelteon, and trazodone. When she was 38, she received a trial of valproic acid for insomnia, compulsion, and explosive behaviors and also tried paroxetine per family request. She also tried olanzapine for increased agitation and hallucinations, which was moderately successful.

**Figure 1:**
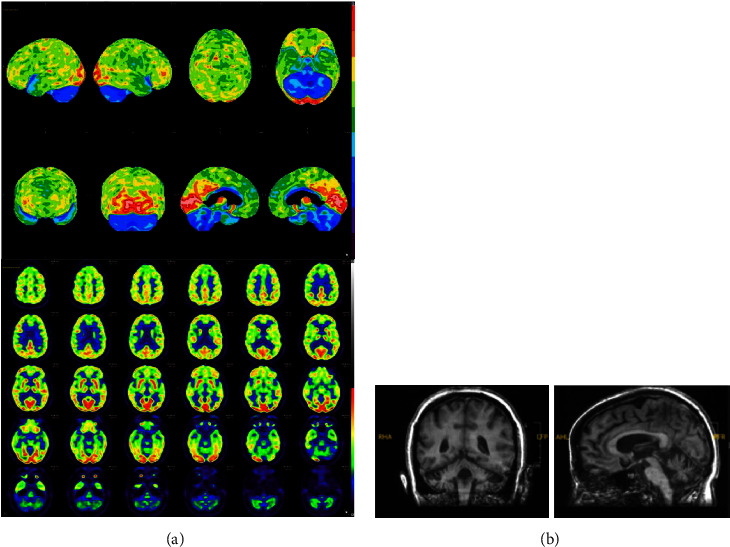
(a) Patient's FDG-PET scan at age 39 showed severe hypometabolism in the cerebellum, medial temporal lobes, paramedial frontal lobes, midbrain, and pons. There was also mild-to-moderate hypometabolism in most of the cerebral cortex except the bilateral occipital lobes, precuneus, and posterior cingulate. (b) Patient's MRI T1 sagittal and coronal scan at age 39 showed cerebellum atrophy.

At the age of 39, the patient had repeat MRI and FDG-PET, which showed interval increase in cerebellar atrophy with increase in hypometabolism in cerebellum, respectively. The metabolic activity in the cerebellum was much less than in the frontal lobe implicating that her symptom of executive dysfunction was likely due to cerebellar dysfunction rather than frontal lobe dysfunction. Additionally, an updated neuropsychological evaluation was completed with the patient engaged in the entire proceeding, including measures that were not previously performed due to emotional and behavioral dysregulation. Compared to her exam at age 37, her cognitive performance was grossly stable with improvement in speeded measure of fine motor dexterity, initial learning performance, and semantic verbal memory but showed possible reduction in working memory and executive functioning. She demonstrated prominent features suggestive of cerebellar dysfunction including difficulty with alternating hand movement and motor sequencing (greater with left vs. right) and gait with short tentative steps and apparent balance weakness. These findings raised the working diagnosis of CCAS considering her decline in cerebellar functioning and coordination, cognitive executive functioning, and affective problems including issues with mood regulation and anxiety, in addition to findings on imaging (Figure 1). The patient completed the Schmahmann CCAS scale, on which she failed five domains (semantic fluency, 14 (passing score (PS): 16); category switching, 6 (PS: 10); digit span forward, 5 (PS: 6); digit span backward, 3 (PS: 4); and verbal recall, 9 (PS:11)). Her total score was 73, placing her in the definite CCAS category.

## 3. Discussion

Based on cerebellar, affective, and subcortical cognitive examination findings, our diagnosis is CCAS. This condition was first proposed by Dr. Schmahmann, and it is characterized by disturbances of executive function, impaired spatial cognition, personality change, affect regulation, and linguistic processing difficulties, which this patient demonstrates. Anatomical and imaging studies have shown the cerebellum to have connection with the prefrontal cortex [2]. CCAS is a widely underdiagnosed disease, partially due to the overlap in symptoms that it shares with other neuropsychiatric conditions. Prior to the development of a sensitive CCAS scale, diagnosis had relied on extensive neuropsychological testing. Schmahmann used his analysis of patients with cerebellar diseases to develop a sensitive CCAS scale including tests of the functioning most affected by the disease, including spatial attention and affective dysregulation [3]. The role of the cerebellum in a variety of neuropsychiatric conditions has been gaining increased recognition, much due to the discovery of the involvement of the C9orf72 gene. Studies have found that expansions in the hexanucleotide repeat in the C9orf72 gene involve the cerebellum in patients with neurodegenerative diseases such as bvFTD [4, 5]. In a large cohort of patients with this mutation, anxiety and agitated behavior were found to be early symptoms, in addition to deficits in the dominant parietal lobe and deficits in episodic memory [4]. The C9orf72 mutation has begun to demonstrate the importance of the cerebellum in connecting the complexity of disease presentation with the underlying brain pathways.

In addition to the role of the C9orf72 mutation, a systematic review demonstrated the specific involvement of the cerebellum in neurodegenerative disorders, unrelated to the C9orf72 mutation. It found that both Alzheimer's disease and bvFTD are associated with significant atrophy in the cerebellum. While patients with Alzheimer's disease showed significant atrophy in the bilateral Crus I, patients with bvFTD showed atrophy in the anterior and superior portions of the cerebellum, in lobule VI, with atrophy being overall more severe in Alzheimer's disease. The study demonstrated distinct patterns of atrophy in the cerebellum of patients with neurodegenerative syndromes historically thought of as primarily involving the cerebral cortex, suggesting many shared connections between the cerebral cortex and cerebellar cortex, and demonstrating the importance of the cerebellum [6].

Cerebellum changes are found in patients with bipolar disorder, as well. Neuroimaging and molecular studies that examine the cerebellum in bipolar disorder found that patients with bipolar disorder have smaller total cerebellar size compared to healthy control, and there is also reduced transcription factor specificity protein 4 (SP4) and brain-derived neurotrophic factor high-affinity receptor tyrosine kinase B (TrkB) in the cerebellum [7]. Another disease in which the involvement of the cerebellum is controversial is schizophrenia. One of the earliest known findings in schizophrenia is ventricular enlargement and cerebral atrophy [8, 9]. Less is known regarding the involvement of the cerebellum in schizophrenia; however, studies suggesting cerebellar abnormalities in schizophrenia have been accumulating over the past few decades. For example, patients with schizophrenia have been shown to have decreased blood flow in the cerebellum, though its role remains controversial [10]. Similar to schizophrenia, studies have also been investigating the involvement of the cerebellum in other disorders such as autism. For example, studies have shown that children with autism have abnormal growth patterns, particularly an increase in volume, in the cerebellum in addition to the cerebrum in early life [11, 12].

This patient provides a great example of the complexity of diagnosis in patients with atypical symptoms of cognitive impairment. She has many symptoms mimicking psychological, neurological, and cognitive diseases, and it took many years and a multitude of testing to come to one diagnosis. Altogether, this case report raises awareness to not take symptoms at face level; further imaging and diagnostic workup is often required in unusual presentations of neuropsychiatric conditions. This case suggests that the role of the cerebellum in major neurocognitive disease does not receive the attention that it deserves and illustrates the importance of expanding our knowledge of the cerebellum and considering the cerebellum as a possible etiology of frontal subcortical cognitive impairment.

## Data Availability

No underlying data was collected or produced in this study.
